# Postmenopausal mild hirsutism and hyperandrogenemia due to granulosa cell tumor of the ovary: a case report

**DOI:** 10.1186/s13256-017-1411-3

**Published:** 2017-08-30

**Authors:** Mulat Adefris, Elfalet Fekadu

**Affiliations:** Department of Obstetrics and Gynecology, University of Gondar, Gondar, Ethiopia

**Keywords:** Granulosa cell tumor, Hirsutism, Virilization, Hyperandrogenemia

## Abstract

**Background:**

Among classes of ovarian tumor, granulosa cell tumors are the least common. In approximately 10% of cases of granulosa cell tumor, androgen will be secreted which will present with hirsutism and hyperandrogenemia. We describe a woman with ovarian granulosa cell tumor who presented with hirsutism.

**Case presentation:**

A 50-year-old woman of Amhara ethnicity, para III, abortion I (induced), presented with excessive hair on her face and lower abdomen of 4 years’ duration which affected her quality of life. Her menopause started 7 years ago. Her body mass index was 29.8 kg/m^2^. She had hair on her upper lip, chin, and lower abdomen; she had a Ferriman–Gallwey score of 10. A pelvic examination revealed that her uterus was of normal size and there was no adnexal mass. Ultrasound finding: her right ovary measured 5 × 4 cm. Her serum testosterone was 254 ng/dl; she was counseled to undergo an exploratory laparotomy but she declined. She presented to our out-patient department 10 months later with a complaint of excessive vaginal bleeding of 18 days’ duration. A sonographic evaluation showed a 12 by 15 cm right adnexal cystic mass. With preoperative diagnosis of testosterone-producing sex cord–stromal tumor of the ovary, an exploratory laparotomy was performed. The laparotomy revealed a 20 by 30 cm right ovarian mass with pathology result of adult granulosa cell tumor.

**Conclusion:**

In postmenopausal women with new hirsutism that is severe or rapidly progressive, the possibility of an androgen-secreting tumor must be suspected and a thorough evaluation is needed before initiating treatment for idiopathic hirsutism.

## Background

Among the major classes of ovarian tumors, sex cord–stromal tumors are the least common [1, 2]. Granulosa cell tumor (GCT) is a sex cord–stromal tumor of the gonads that is classified into two types: the adult-type GCT (AGCT) and the juvenile type. GCT arises far more commonly in the ovaries but on rare occasions it may arise from the testes [3–5]. AGCT accounts for 95% of all GCTs and is usually seen in postmenopausal women, with a median age at diagnosis of 52 years. Approximately 70% of these tumors are hormonally active, and a majority of them secrete estrogen, with 15% being hormonally inert and 10% that can produce androgen [2, 6]. GCT is the most common of the estrogen-producing neoplasms in females and is found to produce estradiol in approximately 40 to 60% of patients [7, 8]. Rarely, a patient may present with virilizing symptoms such as acne, hirsutism, deepening of voice, and clitoral enlargement. This is due to testosterone and/or androstenedione production in a minority of these tumors [5, 9]. To date, few cases of virilizing GCT have been reported in the literature [3]. Our patient was diagnosed as having AGCT yet her initial presentation was with hirsutism and hyperandrogenism; this is one of the rarest presentations of AGCT in postmenopausal women.

## Case presentation

A 50-year-old woman of Amhara ethnicity, para III, abortion I (induced), presented to our out-patient department with the complaint of excessive hair on her face of 4 years’ duration. The hair growth was on her face and lower abdomen; it had gradually increased in amount despite frequent shaving. Her menopause started 7 years ago. She had no vaginal bleeding or discharge; she had no abdominal or pelvic pain. She did her daily chores normally as before but the hair growth over her face affected the quality of her personal life and work life.

**Fig. 1:**
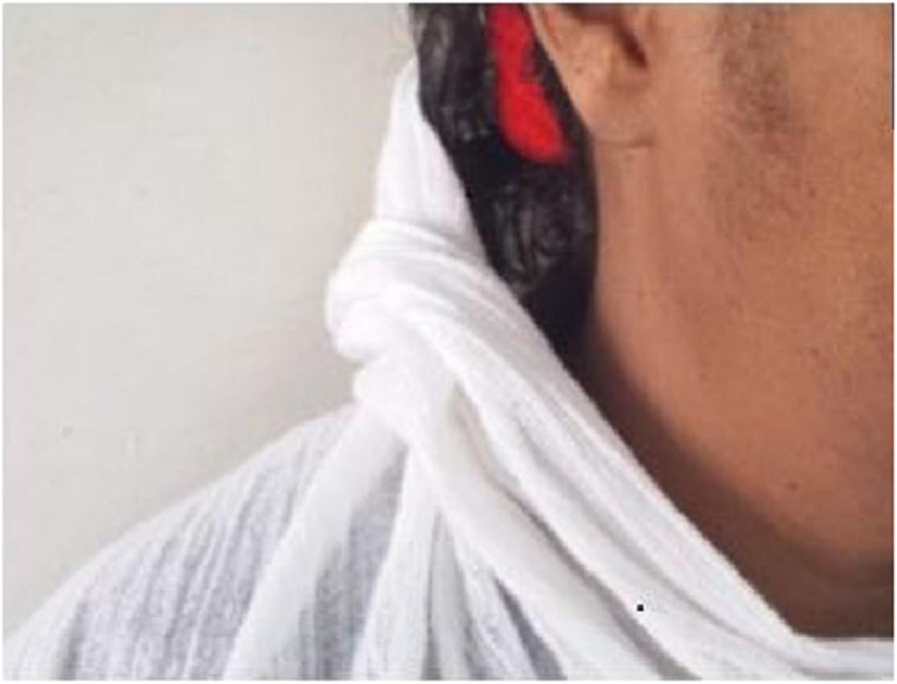
The patient with hirsutism over her face preoperatively

**Fig. 2:**
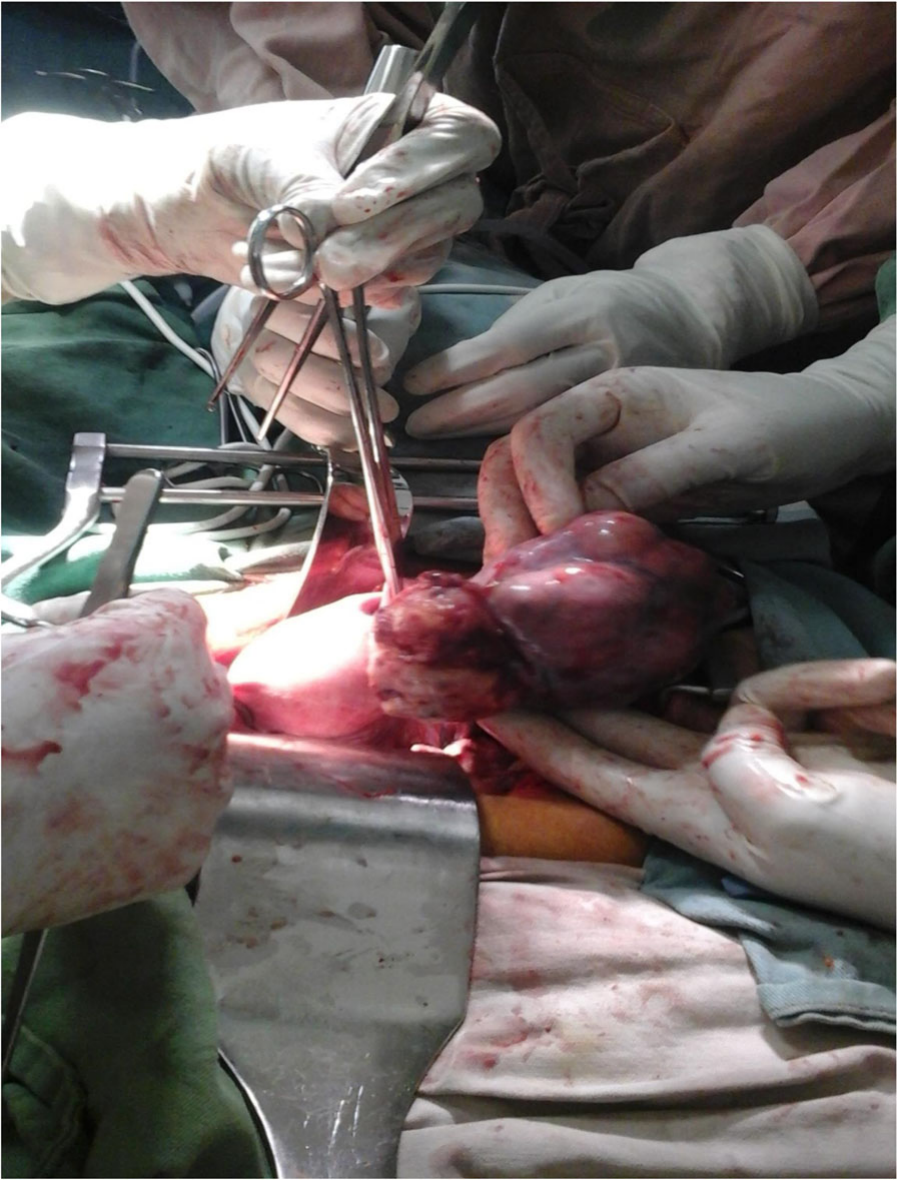
Gross view of the mass intraoperatively

Her weight was 65 kg with a body mass index (BMI) of 29.8 kg/m^2^. Her vital signs were in the normal range and there were no abnormal findings from tests of her chest and cardiovascular system. She had hair on her upper lip, chin, and lower abdomen with a modified Ferriman–Gallwey score of 10, which is mild hirsutism (Fig. 1). An abdominal examination revealed no abnormality. On pelvic examination, her uterus was normal sized. Since she was obese we failed to detect an adnexal mass that was later detected by ultrasound; her right ovary measured 5 × 4 cm. Her left ovary was normal and no ascites or cul-de-sac fluid was seen. No signs of virilization were found in the form of clitoromegaly, frontal baldness, loss of female body contours, increased muscularity, or atrophy of the breast. She had normal complete blood count, electrolytes, and renal and liver function tests. However, her low-density lipoprotein (LDL) and total cholesterol were raised. Hormonal assays revealed: elevated serum testosterone level, 254 ng/dl; normal cancer antigen (CA) 125, 12.65 u/l; and follicle-stimulating hormone (FSH) was 0.74 miu/ml. Other tests for tumor markers and computed tomography (CT) were not performed as they were not available in our hospital. An endometrial biopsy was done and the result was inconclusive. She was counseled to undergo exploratory laparotomy but she declined. After 10 months, she presented to our out-patient department with a complaint of vaginal bleeding of 18 days’ duration, which was minimal initially and subsequently became excessive with dark clotted blood. She does not have a family history of breast or ovarian cancer. She had stable vital signs and upon pelvic examination a 10 by 15 cm right adnexal non-tender, firm, mobile mass was detected. Sonographic evaluation revealed a 12 by 15 cm right adnexal cystic mass with echo debris, thick walled, and it had minimal cul-de-sac fluid collection which was consistent with malignant ovarian tumor. An exploratory laparotomy was performed with a finding of 400 ml ascites and a 20 by 30 cm right ovarian mass with ruptured capsule. Her left ovary and uterus were normal; there was no seeding into peritoneum, omentum, or other intra-abdominal organs. A total abdominal hysterectomy, bilateral salpingo-oophorectomy, resection of the mass, infracolic omentectomy, and right pelvic lymph node sampling was performed and a specimen sent for histopathology (Fig. 2). The result of pathology was AGCT (section from the ovarian mass showed solid insular trabecular patterns and microfollicles formed by proliferation of round to spindle-shaped cells having coffee bean-appearing nuclear grooves and inconspicuous nucleoli in fibrocollagenous stroma, frequent mitotic figures were seen) with normal appearing uterus and cervix, and reactive sinus histiocytosis was seen in the lymph nodes. Her postoperative course was uneventful, her hematocrit was 38%, and she was discharged on postoperative day 5. She was followed-up every month for the first 3 months and then every 3 months. She took five cycles of chemotherapy at a medical oncology unit: bleomycin, etoposide, and cisplatin (BEP). She had more than seven visits after the surgery and chemotherapy. All investigations including complete blood count, liver function tests, and renal function tests were normal. She has no abdominal or pelvic pain, and neither does she have a bowel complaint or a urinary complaint. The hair growth is significantly reduced from before and she does not need to shave her facial hair. Her physical performance is good and she is able to do her daily activities as before. An ultrasound was done once during her follow-up and was normal.

## Discussion

Hirsutism is defined as the presence of terminal coarse hairs in females in a male-like distribution and it affects approximately 5 to 10% of women [1]. The most common cause of hirsutism in women is polycystic ovary syndrome, which accounts for 72 to 82% of cases, whereas androgen-secreting tumors are rare causes of hirsutism in women, comprising 0.2% [7]. Moreover, the most common endocrine manifestation of GCT in postmenopausal women is abnormal uterine bleeding with risk of endometrial hyperplasia and adenocarcinoma reaching 30 to 50% and 8 to 33% respectively [7, 8, 10]. The unusual feature of our patient was that she presented with hirsutism and hyperandrogenemia with a histologic finding of AGCT, for which the majority of cases are estrogenic and commonly manifest with abnormal uterine bleeding or with pelvic mass and pain. The hirsutism was mild which is not a common feature of androgen-secreting ovarian tumor; cases of androgen-secreting ovarian tumor usually have a severe form of hirsutism and/or virilization [10, 11]. Most mild hirsutism is associated with familial or idiopathic hirsutism [1, 10]. But the presentation of this patient was with mild hirsutism that was later found to be hormonally active GCT. Nowadays the severity of hirsutism is determined by the influence it has on a patient’s quality of life, termed “patient important hirsutism,” instead of using the Ferriman–Gallwey score alone to grade the severity [1]. Our patient had significant hirsutism that affected her quality of life.

Similarly, sex cord–stromal ovarian neoplasms are rare; the overall incidence of these neoplasms is 0.2 per 100,000 women. Malignant sex cord–stromal neoplasms are often diagnosed at an early stage, at diagnosis 90% of them are stage I, like in our patient; our patient was staged surgically as International Federation of Gynecology and Obstetrics (FIGO) IC which indicates the tumor was confined to her right ovary with ruptured capsule, but no seeding into her peritoneum, omentum, or other intra-abdominal organs and ascetic fluid was free of malignant cells. However, since outcome is less favorable with stage I disease in which the tumor has ruptured, our patient took five cycles of BEP [5, 7, 8]. Although the diagnosis of GCT may be suspected preoperatively based on the presence of an adnexal mass combined with signs of estrogen or androgen excess or elevated levels of serum tumor markers, the specific tumor type and classification into benign or malignant can be confirmed only with histologic evaluation of the ovary. In our patient, inhibin and other tumor markers were not determined as they are not available in our hospital. The diagnosis was confirmed with histology where granulosa cell of adult type was identified as a solid insular trabecular pattern with coffee-bean nuclei with frequent mitosis. Call–Exner bodies were not mentioned in the pathology report [7].

For a young patient desiring fertility, unilateral conservative surgery is appropriate. However, for a 50-year-old woman, like our patient, a total abdominal hysterectomy with bilateral salpingo-oophorectomy may be preferred even if the mass seems benign and unilateral.

One notable difference between sex cord–stromal neoplasm and other ovarian neoplasms is that lymph node metastases are rare so performing pelvic and para-aortic lymphadenectomy may not be necessary. Nodes should be palpated; lymphadenectomy is required for women with palpable nodal enlargement. Pelvic and para-aortic lymphadenectomy should also be performed in women in whom there is a suspicion of a different histologic type of ovarian malignancy [7]. Our patient presented with features of androgen excess which is a rare presentation of GCT of the ovaries.

## Conclusion

In postmenopausal women with new hirsutism that is severe or rapidly progressive, the possibility of an androgen-secreting tumor must be suspected and a thorough evaluation is needed before initiating treatment for idiopathic hirsutism.

## Abbreviations

AGCT: Adult-type granulosa cell tumor
BEP: Bleomycin, etoposide, cisplatin
BMI: Body mass index
CA: Cancer antigen
CT: Computed tomography
FIGO: International Federation of Gynecology and Obstetrics
FSH: Follicle-stimulating hormone
GCT: Granulosa cell tumor
LDL: Low-density lipoprotein

## References

[1] Choudhary SV, *et al.* Hirsutism with virilization in a postmenopausal woman due to a rare ovarian steroid cell tumor. Indian J Dermatol Venereol Leprol. 2010;76(2):216. 10.4103/0378-6323.60546 20228571

[2] Markopoulos MC, *et al.* Management of endocrine disease: hyperandrogenism after menopause. Eur J Endocrinol. 2015;172(2):R79–91. 10.1530/EJE-14-0468 25225480

[3] González-Díaz E, *et al.* Tumor de células de la granulosa tipo adulto de ovario. Progresos de Obstetricia y Ginecología. 2008;51(10):593–604.

[4] Miller BE, *et al.* Prognostic factors in adult granulosa cell tumor of the ovary. Cancer. 1997;79(10):1951–5. 10.1002/(sici)1097-0142(19970515)79:10<1951::aid-cncr16>3.0.co;2-u 9149022

[5] Sehouli J, *et al.* Granulosa cell tumor of the ovary: 10 years follow-up data of 65 patients. Anticancer Res. 2004;24(2C):1223–30. 15154651

[6] Kota SK, *et al.* Ovarian granulosa cell tumor: an uncommon presentation with primary amenorrhea and virilization in a pubertal girl. Indian J Endocrinol Metab. 2012;16(5):836. 10.4103/2230-8210.100658 PMC3475918 23087878

[7] Barakat RR, Markman M, Randall M. Principles and practice of gynecologic oncology. Philadelphia: Lippincott Williams & Wilkins; 2009.

[8] Pant P, *et al.* Granulosa cell tumors. J Inst Med. 2009;30(2):49–51.

[9] Vera L, *et al.* Increasing hirsutism due to a granulosa-cell tumor in a woman with polycystic ovary syndrome: case report and review of the literature. Gynecol Endocrinol. 2013;29(4):273–7. 10.3109/09513590.2012.743012 23327624

[10] Bahadir C, *et al.* Postmenopausal hirsutism and hyperandrogenemia due to granulosa cell tumor of the ovary. 2013.

[11] Önderoglu LS, *et al.* Bilateral ovarian fibromatosis presenting with ascites and hirsutism. Gynecol Oncol. 2004;94(1):223–5. 10.1016/j.ygyno.2004.03.042 15262147

